# Exploring attributes of high-quality clinical supervision in general practice through interviews with peer-recognised GP supervisors

**DOI:** 10.1186/s12909-021-02882-7

**Published:** 2021-08-20

**Authors:** Belinda O’Sullivan, Helen Hickson, Rebecca Kippen, Glen Wallace

**Affiliations:** 1 General Practice Supervisors Australia, PO Box 141, Bendigo North, Victoria 3550 Australia; 2 Rural Clinical School, Faculty of Medicine, University of Queensland, Locked Bag 9009, Toowoomba, Queensland 4350 Australia; 3 School of Rural Health, Monash University, PO Box 666, Bendigo, Victoria 3550 Australia; 4 La Trobe Rural Health School, College of Science, Health and Engineering, La Trobe University, Bendigo, Victoria 3550 Australia

**Keywords:** Supervision, Quality, General practice, Physician, Education, Learning, Feedback, Teaching

## Abstract

**Background:**

Clinical supervision in general practice is critical for enabling registrars (GP trainees) to provide safe medical care, develop skills and enjoy primary care careers. However, this largely depends on the quality of supervision provided. There has been limited research describing what encompasses quality within GP clinical supervision, making it difficult to promote best practice. This study aimed to explore the attributes of high-quality clinical supervision for GP registrars.

**Methods:**

In 2019–20, 22 semi-structured interviews were conducted with GP supervisors who were peer-nominated as best practice supervisors, by Regional GP Training Organisations and GP Colleges in Australia. Purposeful sampling sought respondents with diverse characteristics including gender and career stage, practice size, state/territory and rurality. Interviews were conducted by video-consultation and recorded. De-identified transcripts were independently coded using iterative, inductive thematic analyses to derive themes that reflected quality in GP supervision.

**Results:**

Seven themes emerged. Participants understood the meaning of quality supervision based on their experience of being supervised when they were a registrar, and from reflecting and learning from other supervisors and their own supervision experiences. Quality was reflected by actively structuring GP placements to optimise all possible learning opportunities, building a secure and caring relationship with registrars as the basis for handling challenging situations such as registrar mistakes. Quality also encompassed sustaining and enhancing registrar learning by drawing on the input of the whole practice team who had different skills and supervision approaches. Strong learner-centred approaches were used, where supervisors adjusted support and intervention in real-time, as registrar competence emerged in different areas. Quality also involved building the registrar’s professional identity and capabilities for safe and independent decision-making and encouraging registrars to reflect on situations before giving quality feedback, to drive learning.

**Conclusions:**

This study, although exploratory, provides a foundation for understanding the quality of clinical supervision in general practice, from the perspective of peer-recognised GP supervisors. Understanding and adopting quality within GP supervision may be improved by GPs sharing exemplars of best practice and having opportunities for professional reflection. The findings could be used as a point of reference for devising GP supervisor curriculum, resources and professional development activities.

## Background

Clinical supervision of doctors learning to be general practitioners (called GP registrars) is critical for enabling safe medical care and producing skilled doctors who are invested in primary care careers. However, to some extent this relies on the quality of supervision that GP registrars are provided. Quality variation is a major issue within general practice training, as registrars (GP trainees) learn in distributed regions and GP practices that may have widely differing perspectives of optimal supervision. Understanding the dimensions of quality supervision may provide insights for planning, benchmarking, and supporting optimal supervision practice.

This study is based in Australia, as a case study of a country undertaking medical workforce policy reforms aiming to produce more general practitioners [1, 2]. Policy reforms include a strong focus on improving the quality of general practice training, to attract people to GP training and produce skilled doctors. Like many countries, Australia requires a higher proportion of GPs for the increasing demands for chronic and complex care in the community, including as the population ages [3]. In Australia, there are approximately 5200 GP registrars spanning one of 3–4 years of vocational training and aiming to pass national College assessments to achieve fellowship [2]. GP registrars are employed by the practices that they train in and work under the guidance of a clinical supervisor as they learn to deliver a wide scope of primary care services including for vulnerable and high-risk groups in the community [4]. GP supervisors are considered fellowed GPs who have completed—or are completing—a supervisor orientation course with a Regional Training Organisation (RTO; one of nine in Australia). They must also agree to deliver on a range of broad functions as part of training practice accreditation [5, 6] (Table 1). Despite the minimum accreditation requirements, there is still no formalised national curriculum, nor quality framework for GP supervision work in Australia.

**Table 1:** Broad requirements and functions of GP supervisors in Australia [5, 6]

• Have requisite experience as a doctor (varies but 4–5 years is a benchmark, and may include the supervisor’s own general practice training of 3–4 years)	
• Understand training requirements and breadth of scope of knowledge, skills and experience that are required of the registrar (GP trainee)	
• Understand the type of supervision that is required of the registrar (noting their training stage and college of GP training that they are enrolled in)	
• Negotiate methods and frequency of communication with the registrar	
• Meet with the registrar early in the placement to discuss and appraise the registrar’s skills and experience and develop a learning plan	
• Provide appraisal and formative assessment of the registrar in accordance with their stage of learning	
• Provide or facilitate structured educational activity requirements according to the registrar’s training stage and experience	
• Organise their own clinical workload to be compatible with the teaching commitments	
• Ensure the number of registrars under supervision does not exceed supervisor’s ability to provide effective supervision	
• Ensure that another supervisor is available when they are not available	
• Participate in supervisor training and other activities to gain accreditation and develop skills	
• Be an excellent role model	

Informing quality within GP supervision is important to assist with standardising GP training, where multiple GPs in the one practice may supervise registrars in different ways and registrars also rotate to at least two practices during their GP training. Where GPs have a different understanding of supervision quality, there is a high potential for registrars to have a negative training experience at some point in their training cycle. Quality standards may assist registrars to receive more continuity of optimal learning, aiding their enjoyment of general practice at the formative stages of their career and helping them to pass assessments and achieve fellowship. The community may also gain from consistent best practice supervision as this enables access to the full suite of safe primary health services from any registrars with whom they may consult.

Multiple groups have a stake in engaging and training GP supervisors in the Australian context. General Practice Supervisors Australia (GPSA) is a peak body that leads the development of up-skilling resources for GP supervisors, under the Australian General Practice Training Program (AGPT). GPSA delivers supervisor-led webinars, guides, and teaching plans year-round. Additionally, the RTOs and GP Colleges engage in supervisor’s professional development courses and networking events. However, across the nation, supervision resources have emerged informally and iteratively, without a strong holistic understanding of quality within GP supervision.

The existing literature mainly describes the GP supervisor’s role, rather than the attributes of quality supervision. An integrative literature review suggested that the GP supervisor role is multi-faceted [4]. They establish learning environments, assess learning needs, facilitate learning, monitor the content and process of learning and registrar well-being as they guide registrars from ‘know that’ to ‘know how’ [4]. Another narrative overview suggested that supervision roles may vary by the registrar and practice context such as rurality and part-time work [7]. GP supervisors also undertake regular communication, conflict resolution, clinical reasoning, critical thinking [8] and support for registrars who are struggling [9, 10]. Another literature review identified the relationship between the GP supervisors and registrars is important along with giving clear feedback and allowing registrars to have input into supervision processes [11]. However, there is a need for more research to understand how quality is represented by the interplay of various functions, so that supervisor professional development an be anchored to a best practice framework [12]. There is also room to link the concept of quality supervision to vocational learning theories.

The aim of this study was to explore the attributes of high-quality clinical supervision for registrars in general practice.

## Methods

This study applied a qualitative descriptive approach [13] involving in-depth, semi-structured interviews. The study recruited GP supervisors Australia-wide, who had been peer nominated as best practice supervisors. This group was chosen as they were industry-recognised for supervising and therefore considered suitable for informing quality within supervision practice. Ethics approval for the study was obtained from the Monash University Human Research Ethics Committee (Project ID 21655), ratified at the University of Queensland (Project ID 2012001171).

### Procedure and semi-structured interviews

The study was led by GPSA, at the interest of the GPSA board, to inform the directions for quality improvement of supervision. In March 2019, participants were recruited via the nine RTOs, the Royal Australian College of General Practitioners (RACGP) and the Australian College of Rural and Remote Medicine (ACRRM), to ensure a breadth of national representation. These organisations were sent information about the study and invited to contact GPs in their networks, who they considered best practice supervisors, including recipients of their own ‘Supervisor of the Year’ awards over the last 10 years. GP supervisors with a mix of characteristics such as gender and career stage, practice size, and location in different states/territories and ruralities were sought, to include a range of perspectives from different contexts.

Only one reminder was issued, as the study was well-subscribed. The sampling frame included 60 GP supervisors. After contacting this group, 22 supervisors with a broad range of characteristics, completed the enrolment and participated in the study. Participants received an $AUD150 gift card in recognition of their time. Recruitment ceased on a practical basis in April 2020, as the COVID-19 pandemic had caused a diversion of time and resources in general practices which made it too busy for GP supervisors to respond [14].

After completing written informed consent, 22 recorded video-conference semi-structured interviews of 50–70 min duration were conducted between September 2019 and June 2020. The interviewer was an experienced PhD-trained qualitative researcher, not known to the participants, who was a trained clinician in a non-medical field. The interviewer had experience of working with general practices in selected regions related to coordinating primary care projects. The interview duration was participant-driven and guided by a schedule (Table 2), which had been informed by the research team and piloted with two experienced GP supervisors. During interviews, participants were asked to describe their supervision experience, practice context and aspects of their supervision that they considered reflected high quality. Each interview was transcribed verbatim, linked by a unique identifier and then de-identified before being circulated to the research team for analysis. Secondary data collection included interview notes/reflections and minutes of research meetings for the purpose of self-reflexivity and transparency about any of the research challenges, in seeking to achieve the research aim [15].

**Table 2:** Interview Schedule

Question	Prompt
1. Firstly can you tell me a bit about yourself and your practice?	
2. Can you please tell us a bit about your experience with supervising registrars?	
3. When did you start supervising and how was it that you started?	Choice, landed in it incidentally, did you always want to do it? Why did you start supervising?
4. What sets you apart from other GP supervisors?	Can you think of colleagues who you think are/were good supervisors and reflect on your own practice that way?
5. What is your approach to supervising?	What makes it good quality - prompt try to pin down to skills that could be trained, rather than just personality factors?
6. How did you learn this approach? Did you use any specific resources?	Nothing I just did it, reflective practice/experiential learning, off the job training or specific professional development that they might have done, observing professional colleagues. Were any of the resources particularly useful?
7. Does your general practice impact the quality of your supervision in any way? How?	The way the practice is set up, the colleagues, patients. What is the ideal general practice environment for high quality supervision? Contrast any experience of changing practices on supervision work, any impact, also rural versus metropolitan.
8. To what extent do any registrar attributes impact on the quality of your supervision? How?	Different types of registrars Contrast the impact of different registrars, adapting to different learner styles, personalities, specific issues.
9. Was there ever a time you felt like quitting the supervision role? Tell me about it.	Things that left you feeling burnt out or where the gain wasn’t worth the effort, why didn’t you quit and what helped?
10. How do you think supervision of registrars changed over the course of your career?	Any impact on the types of skills needed to be an effective supervisor. Have registrars changed over time, and why?
11. What do you think are the skills that contemporary supervisors need?	

### Data analyses

The analyses were informed by theories of work-based learning suggesting that the conceptualisation of quality supervision might occur naturally in the workplace, through a multiplicity of opportunities and interactions [16]. Additionally, theory about the quality of vocational education was used, where quality teaching/supervision evolves from the intersections of knowledge and professional culture, specific to the institutional environment in which they practice [17].

Whilst considering these theories, the research team sought to interpret emergent findings in the data. As such, analyses commenced with three members of the research team reading the full transcripts and independently coding the data. This happened with no pre-set coding frame, in line with inductive analyses processes [18]. Additions and alterations to the codes were made as blocks of five transcripts were completed. Authors then double-coded another transcript identifying reasonable concurrence with the codes and adding extra codes if these were relevant. The material was discussed, annotated, and then organised into emerging themes, layering and reorganising these to make sense of the data [19]. This first stage of analyses occurred with the research team working in distributed sites, and meeting online, fortnightly. The team regularly challenged each other’s ideas, to reduce subjective biases and test any assumptions [15].

The research team then attended a half-day face-to-face analysis session to allow whiteboarding of concepts around the emerging themes and to dig deeper into the themes. The broad general practice supervision requirements (Table 1), and other national clinical supervision competencies [20] were used to explore concurrence. Based on this, it was found that the inductive themes provided a richer perspective of quality GP supervision, and used language and concepts specific to the general practice vocational learning context. The research team pursued further rounds of inductive analyses by re-reading the original transcripts for meaning about quality and used face-to-face and online discussions to allow for internal confirmation or disconfirmation. This process enabled thick description and triangulation of a final set of themes that the team agreed upon [15].

To aid analyses, participant characteristics were appended to unique identifiers and accompanied all transcript codes and thematic text. These depicted gender and career stage, practice size, state/territory, and rurality, coded according to the Modified Monash Model (MM1 depicting metropolitan areas and MM2-MM7 increasing degrees of rurality) [21].

## Results

Participants of the semi-structured interviews were from a wide range of career stages, practice sizes and locations (Table 3).

**Table 3:** GP and practice characteristics (*n* = 22)

Characteristic	n	%	Characteristic	n	%
***Gender***			***State/Territory***		
Male	16	73	New South Wales / Australian Capital Territory	6	27
Female	6	27			
Total	22	100	Victoria	2	9
***Years post fellowship***			Queensland	4	18
Less than 10 years	7	32	South Australia / Northern Territory	4	18
10 to less than 20 years	5	23	Western Australia	3	14
20 to less than 30 years	6	27	Tasmania	3	14
30 years or more	4	18	Total	22	100
Total	22	100	***Rurality***		
***Practice size***			MM1. Metropolitan area	9	41
3–5 GPs	6	27	MM2–MM4. Regional centre, large or medium rural town	5	23
6–12 GPs	6	27			
13–19 GPs	6	27	MM5–MM7. Small rural town, remote or very remote community	8	36
20 or more GPs	4	18			
Total	22	100	Total	22	100

Despite sampling participants of broad characteristics and exploring variation by career experience, practice or registrar factors, the perspectives of quality supervision were relatively consistent. The data produced 7 main themes (Table 4). Participant quotes were labelled with participant number (P1–P22), gender, and years’ post-fellowship. Further participant-characteristic labels for quotes were avoided to maintain anonymity.

**Table 4:** Themes related to the quality of supervision of GP registrars in general practice

Theme	Description
Understand the meaning of quality supervision and seek to continually refine practice	Having a concept of quality in supervision work, drawing from lived experience as a registrar. Continually refining practice, reflecting and learning from other supervisors and personal experience. Listening to feedback about your supervision from registrars.
Structure placements with a focus on optimising learning	Ensuring a good match between registrar interest and their motivations and what the practice/supervisor can offer. Using resources and doing a holistic orientation to improve understanding of the business. Establishing the foundations for how the learning will happen around the GP business of seeing patients. Establishing learning boundaries, respecting business responsibilities and the availability of other supervisors onsite.
Build secure and caring relationships with registrars	Front-loading support and maintaining the registrar relationship to build the foundations for discussing and coping with uncertainty, mistakes, and difficulties. Showing genuine care about registrar and holistically understanding the registrar to contextualise teaching and learning. Demonstrating vulnerability to promote open lines of communication.
Sustain and enhance learning opportunities drawing from the whole practice team	Ensuring registrars engage with the whole practice team so that supervision is sustainable. Promoting connections to the wider practice, specific to a achieving a breadth of learning goals. Ensuring registrars can access different skills and styles of supervision in the practice team.
Use learner-centred supervision, adjusting the supervision model as required	Tailoring learning to the needs and style of each registrar. Preparing to adjust the teaching or supervision style to suit the learner. Evaluating learning across the breadth of general practice and using structured and opportunistic ways to extend learners.
Build professional identity and foster safe, independent decision-making	Building understanding of working effectively in the general practice context, clinical best practice and understanding of comprehensive practice. Encouraging registrars to build their own style and achieve independent decision-making, also knowing when to ask for help.
Encourage registrar reflection and give quality feedback to drive learning	Giving feedback on positive and negative aspects of performance even when conversations may be difficult. Giving registrars space to reflect and problem-solve. Gathering perspectives of registrar’s performance from others in the practice as another source of feedback.

### Understand the meaning of quality supervision and seek to continually refine practice

Participants unanimously reported that teaching is a good thing to do, both personally and professionally. However, they found it challenging to talk about the quality of their supervision in a way that could differentiate their approaches from those of colleagues. Through prompting, these supervisors related that they had mostly benchmarked the quality of their supervision in relation to other GP supervisors that they had encountered along their own learning pathway, “*I watched how good supervisors taught me. And I saw how bad supervisors taught me*” [P1: male, < 20 yrs]*.* They noted, “*easily remember[ing] the good things my own supervisors taught me and told me. So, I know in my teaching sessions with my registrars, I often reference what my own supervisors told me*” [P17: male, < 20 yrs]. Another participant reinforced that having experienced good and bad supervision as a registrar, “*I know which supervision worked...So I try and provide that*” [P4: female, < 20 yrs]*.*

They also observed other GP supervisors, which helped them to reflect on issues related to supervision quality, “…*we often try to take as much of the things that you go, ‘Well, that looks good. That’s a good way of doing something.’ And removing the ways that you go, ‘Oh, that didn’t work so well.’ You try to remove as much as you can for the things don’t work well*” [P18: male, < 20 yrs]*.* They also revisited their teaching tools and resources over time “*from our experiences of what’s helped*” [P4: female, < 20 yrs] and trialled different processes, then reflected about whether they “*worked a little bit better*” [P20: female, < 20 yrs]. When supervising registrars and their strong contemporary knowledge in lots of areas, quality supervisors showed capacity to learn, to “*rethink and reflect whether I’m doing things right, or whether things could be done better and regularly I find something that I change*” [P12: male, 20 + yrs]. Respondents also noted that, they valued “*be[ing] able to take feedback from the registrar. To listen*” [P18: male, < 20 yrs], as part of improving their supervision.

### Structure placements with a focus on optimising learning

Quality was reflected by actively structuring and preparing placements to optimise learning. Emphasis was placed on firstly selecting registrars suited to the practice, by checking on their motivations to be a GP, “*I try and get an idea of why they want to go into general practice. I’m looking for people who actually do want to...truly help their patients*” [P6: male, < 20 yrs]. Another noted the importance of matching clinical skills, “*So some of them might say, ‘I’m really interested in women’s health.’ And I say, ‘Unfortunately, I’m not the person for you’*” [P1: male, < 20 yrs].

Respondents also undertook holistic orientation processes using a suite of resources such as checklists and guides. They introduced registrars to infrastructure and processes, “…*for the first two days...there’s a very formal welcome and tour*…*they sit in with me, watch me see a few patients*...*more about learning the computer than anything else*” [P11: male, 20 + yrs]. Registrars also met with and observed other practice staff, “*meet the reception... see how the bookings are made, how the billings are done…time with the nurses*” [P3: male, < 20 yrs].

Participants also planned to safeguard quality clinical teaching time around seeing their own patients. One noted, “*thinking about how you set up your teaching time…You actually need to timetable that in so that it does happen…and then the sort of curriculum that should be progressed through*” [P19: male, 20 + yrs]*.* Quality supervision was also depicted by clarifying learning boundaries within the GP context where there were business responsibilities and may be limited other supervisors onsite, saying “‘*This is what your background is, this is what your skills are. This is what my background is, this is what my skills are. This is also how we’re going to do it, this is the rostering, this is how you contact me through the day’...it’s all laid out*” [P9: male, < 20 yrs]*.*

### Build secure and caring relationships with registrars

Participants strongly emphasised that quality supervision involved building relationships with registrars. Quality supervisors took leadership of this, aiming to build a secure relationship with registrars at their commencement of a training term. They noted that this involved “*front load[ing] the support in the first couple of weeks*” [P11: male, 20 + yrs] and consolidating the relationship as registrars went through “*difficulties...and...good times*” [P3: male, < 20 yrs]*.*

Supervisors described that the focus of the relationship was on genuine care for the person and their learning, one commenting “*you actually have to care about the learner and want them to be the best clinician or person that they are*” [P5: male, < 20 yrs]*.* Understanding the registrar at a holistic level was considered to enable supervisors to contextualise and guide registrar performance:

*...it works much better if it's a personal relationship. It's especially understanding where they come from? What's going on in their lives? It does impact on how they perform* [P12: male, 20+yrs]*.*

A secure and caring relationship was the enabler of “*talk[ing] about the difficult things…uncertainty, mistakes*” [P2: female, < 20 yrs]. It was also viewed as central to the “wellbeing component to your [supervisor] role... to look out for the whole person, not just their clinical training” [P15: male, < 20 yrs].

Within relationship building, supervisors purposefully aimed to “*get rid of the power imbalance*” [P12: male, 20 + yrs] and maintain open lines of communication*.* They did this by “*role modelling vulnerability*” [P21: male, 20 + yrs] involving showing it is normal “*to ask your colleague for advice and questions and take about mistakes and difficulties*” [P2: female, < 20 yrs]. They drew on key negative events to build trust for open communication:

*Well yes, you did scratch dad's car and he's not happy. Dad’s still going love you tomorrow, and he'll still feed you.…We'll forget about it* [P12: male, 20+yrs]*.*

### Sustain and enhance learning opportunities drawing from the whole practice team

Quality also entailed working sustainably and ensuring registrars gained as much as they could from the learning opportunities available across the wider practice team, with one respondent noting: “*It’s a group thing. You cannot do it alone... You’ve got to work as a team*” [P7: female, 20 + yrs]. Others similarly reflected that this was a way to ensure registrars had enough oversight and support when they were busy with their own patients*:* “*I guess trying to facilitate a team environment because I don’t know how I would do this on my own*” [P4: female, < 20 yrs].

Further, quality supervisors acknowledged their own skills boundaries as well as their expertise in some clinical areas that could add value to registrar learning. They promoted connections that could address a breadth of learning goals:

*...there are people in my practice who are much better at dermatology than I am. So in terms of supervision for formal exposure to stuff, I hand that over to other people* [P8: female, 20+yrs].

Participants also considered that the whole practice team could help registrars to access other styles of general practice and teaching for developing broader perspectives*,* “*there’s about half a dozen supervisors…all very good. We have slightly different styles*” [P9: male, < 20 yrs].

### Use learner-centred supervision, adjusting the supervision model as required

Quality was reflected by tailoring their supervision model to the needs and style of each registrar, through “*spend[ing] quite a bit of time with getting to know what their background is, how they learn, what’s best for them, and trying to fit that into how we work*” [P5: male, < 20 yrs]*.* Several commented that this involved “*adapt[ing] my teaching*” [P10: male, 20 + yrs] and “*adjust[ing]…supervision style*” [P7: female, 20 + yrs] depending on individual registrars. This included working out the methods that optimised the registrar’s capacity to learn, “…*some people, obviously, like to learn from didactic teaching in which case you can just do a didactic talk. Others... learn a lot better when you have to ask them more questions and do their own research*” [P10: male, 20 + yrs].

Quality also involved evaluating the strengths, weaknesses, clinical interests and motivations of each registrar and addressing these in structured and opportunistic ways during a training term. The breadth of clinical aspects of general practice made this exercise quite extensive:

*And so I said,* ‘*Look we're going to deal with general practice. We're going to start from the baby right up to palliative care and everything that's going to be in between. And we're going to figure out where you're weak, and we're going to make you stronger’* [P1: male, <20yrs].

### Build professional identity and foster safe, independent decision-making

Quality was reflected by supervisors developing a general practice identity including how to work effectively in the unique general practice context, “*it’s... a totally different work environment*…*appointments and reception, billing, prescribing, nursing, pathology, pharmacy*” [P13: male, 20 + yrs]. This included engaging registrars in clinical best practice, “*we work on lots of evidence-based approaches to patient management*” [P19: male, 20 + yrs] and developing knowledge of “*comprehensive general practice*” [P5: male, < 20 yrs]. Given general practice involves doctors working in independent consulting rooms, quality supervisors also noted building registrar confidence about their own style noting, “*everyone practices differently*” [P5: male, < 20 yrs].

Respondents encouraged registrars to think like a GP and learn the balance between independent decision-making and asking for help when they needed it. They spent time understanding “*how secure the registrar is in decision-making, and how much trust the supervisor in the practice can have in the medical assessments made by that registrar*” [P8: female, 20 + yrs].

### Encourage registrar reflection and give quality feedback to drive learning

Respondents noted that a “*key and critical thing is being able to give... quality feedback... about something done well or feedback about something that needs improvement*” [P14: female, < 20 yrs] whilst acknowledging that this could be “*challenging*” and “*one of the hardest things [to do]*” [P4: female, < 20 yrs].

Giving constructive feedback also involved seeking registrar insights, “*inviting a conversation into,* ‘*How do you think it went in there? What went well, what went badly?’*…*giving them a chance to give an account and then give your account*” [P6: male, < 20 yrs]. They spoke about taking an “*encouraging*” stance, giving space for registrars “*to come reflect on their own consultation style, their own management process, or their thought process, and see where that may be falling down, and come up with an idea of how they would maybe do it better*” [P20: female, < 20 yrs].

The secure and caring relationship was noted to intersect with this theme, where it enabled the process of giving open and honest feedback, “*a much easier conversation*” [P5: male, < 20 yrs].

Quality supervisors also gathered feedback about the registrar’s performance from other staff in the practice and applied this to support registrar learning:

*When it comes to the actual performance, it's feedback from the staff. The reception at the front will know that the patient is unhappy about doctor X, and they'll tell me. I can feed that back to the registrars* [P3: male, <20yrs].

## Discussion

This exploratory study provides some insights into the concept of quality clinical supervision for GP registrars learning in the general practice context. Quality supervision was understood when GPs reflected on their own supervision experiences and anecdotes from colleagues. This is consistent with work-based learning theory, where quality in supervision is conceptualised through situated, experiential learning, based on real-world exemplars [16]. It also fits with vocational teaching more broadly, where the understanding of quality is strongly tied to what works well in a unique institutional environment [17]. However, GP supervision can be somewhat siloed by its occurrence in distributed regions and general practices, whereby it may be critical to develop practical exemplars and connect GP supervisors professionally to promote cross-learning and support learning about best practice supervision. This could occur by developing GP supervisor “communities of practice”. Communities of practice are known to work well when groups with shared interests mutually guide each other through their understanding of the same problems in their area [22]. Some of the resources that could be also shared in GP supervisor networks are processes and tools, such as orientation checklists, quality self-reflection tools and topic pro-formas.

Quality of supervision encompassed a comprehensive approach where supervisors recognised and drew from a deep understanding of practice and learner contexts to find synergies that would improve registrar engagement in general practice and their performance as an emerging GP. Other narrative literature suggests that an educational alliance between supervisor and learner is important to facilitate registrars doing tasks of increasing complexity [7]. Our findings suggested that supervision quality involved investing in secure relationships as a foundation for registrars to receive rich and potentially challenging feedback, around areas of uncertainty and mistakes. This builds on previous literature reviews that place relationship-building, communication, and feedback at the crux of a supervisor’s role [11]. With a strong relationship and open lines of communication, quality supervision involved taking opportunities to get registrars to reflect on their own performance, even if situations were challenging.

Quality of supervision was also reflected by supervisors who were learner-centred, and who used precise structure and advice to promote optimal learning relative to an emerging set of competencies. This also included helping registrars to become highly functional and conscious of safe, but autonomous decision-making to equip them for their future as an independent GP. This aligns with research suggesting quality in vocational learning encourages learners to take responsibility and feel empowered [23]. Professional identity was also developed by linking learning to models of comprehensive GP work, which supervisors recognised may be new concepts given many registrars enter general practice from a hospital environment that is very different to primary care. Quality supervision meant that skill levels were not assumed but actively screened upon entry to the practice, and regularly monitored, allowing for both remediation and extension in real-time. Supervisors also adjusted their coaching around the registrar’s needs and their capacity for self-regulated learning building on the findings other research [24]. Critically, this suggests that quality supervision is highly dynamic, and the broad requirements of supervisors in Australia (Table 1) may need to better reflect the constant intersecting cycle of activities involved in quality supervision, rather than narrow, or siloed tasks. Further, this research suggests that learning to be a quality supervisor could be challenging as GP supervisors often work in discrete practice settings, and building quality supervision skills may require regular real-life practice with mentorship from experienced GP supervisors.

Within the private business model of general practices, quality in supervision also entailed selecting registrar’s with an attitude, needs/interests which best fit with the practice’s resources. Selection of registrars has not previously been described as a quality issue for supervision but if a registrar is poorly suited to a practice, and there are limited supervisors in the practice, it may be extremely challenging to provide supervision at a level and range that will meet their needs. Another issue for quality within supervising in a private business model was being able to draw other team members into the supervision model to enable supervisors to progress their own clinical caseload. A key implication is that small practices may need to get more capable registrars who are self-directed as the supervisors may otherwise find supervising unsustainable.

Although this study was small and exploratory, it provides the first holistic description about quality within supervision of GP registrars and therefore informs ongoing work to develop optimal general practice training. It may have specific application to quality supervision standards, curriculum, and resources development.

The study also had several limitations. It was based in Australia, and the study should be validated with respect to the context of GP training in other countries. Whilst including GP supervisors who were peer recognised, this is still a subjective measure of their standard of supervision. Interviewing under-performing supervisors may have enhanced the potential for this study to reflect on the quality of supervision practice. It is possible that the interruption of this research by the COVID-19 pandemic affected the saturation of the findings, although there were signs that there was minimal new material arising from the last few interviews. Interpretive validity and trustworthiness was strengthened by verbatim quotation, independent and double-coding, and taking 9 months to explore the themes in depth. All efforts were made to collect the participants’ views in this study, although it is possible that self-disclosure was limited by apprehension of the interviewing researcher’s relationship as an employee of GPSA, or that the participants ideas and expectations of the research impeded discussion.

## Conclusions

This study, although exploratory, provides a starting point for understanding the quality of supervision in general practice from the perspective of GP supervisors who are peer recognised for their supervision work. Quality supervision was understood through lived experience and personal reflection. It encompassed building a caring and trusted relationship with the registrar, drawing on input of the whole practice team, using a learner-centred models and adjusting input in real-time as registrar competency emerged. Quality was also depicted by building registrar professional identity and capabilities for safe and independent decision making whilst promoting regular reflection and feedback processes, even when issues were challenging. The findings may be applied to inform quality supervision standards and resources. Professional networks that link GP supervisor for sharing practical exemplars and resources may improve the capacity to conceptualise and embed quality supervision in general practice.

## Data Availability

The data supporting the conclusions of this article are available to researchers upon request, subject to ethical approval.
